# Supplementary material for: COVID-19 vaccination and major cardiovascular and haematological adverse events in Abu Dhabi: retrospective cohort study
Source: Nat Commun. 2024 Jun 28;15:5490. doi: 10.1038/s41467-024-49744-6 (PMC11214614; doi:10.1038/s41467-024-49744-6)
Supplement: Supplementary file 1 — Supplementary Information [file 41467_2024_49744_MOESM1_ESM.docx]

**Supplementary Information**

**Title**

COVID-19 vaccination and major cardiovascular and haemotological adverse events in Abu Dhabi: retrospective cohort study

**Authors & Affiliations**

Marco A. F. Pimentel ^1^, Maaz Shaikh ^1^, Muna Al Safi ^2^, Yousuf Naqvi ^2^, Shadab Khan ^1^

^1^ M42, Abu Dhabi, UAE. ^2^ Department of Health (DOH), Abu Dhabi, UAE.

| Outcome of interest | ICD-10 Code(s) |
| --- | --- |
| Non-hemorrhagic stroke (NHS) | G45.8, G45.9, I63.* |
| Hemorrhagic stroke (HS) | I60.*, I61.*, I62.* |
| Acute myocardial infarction (AMI) | I21.* |
| Myocarditis/pericarditis | B33.22, B33.23, I30.*, I40.* |
| Pulmonary embolism (PE) | I26.* |
| Disseminated intravascular coagulation (DIC) | D65 |
| Venous thromboembolism (VTE) | I26.*, I82.210, I82.220, I82.290, I82.3, I82.4*, I82.6*, I82.A1*, I82.B1*, I82.C1*, I82.81*, I82.890, I82.90 |

**Table S1. Outcomes of interest and corresponding ICD-10 codes**

**Table S2. IRR (incidence rate ratio) and 95% CI by age group (aged under 40 years, and 40 years or older) and gender (male and female) for the primary outcome in the predefined risk interval of 1-21 days after exposure to vaccination. CI: confidence interval; pdays: person-days.**

|  | **Female** | | | **Male** | | |
| --- | --- | --- | --- | --- | --- | --- |
|  | **Events in risk interval per 1M pdays (n)** | **Events in comparison interval per 1M pdays (n)** | **IRR (95% CI)** | **Events in risk interval per 1M pdays (n)** | **Events in comparison interval per 1M pdays (n)** | **IRR (95% CI)** |
| **Either vaccine** | |  |  |  |  |  |
| Dose 1 | 9·81 (38) | 12·89 (74) | 0·74 (0·48 to 1·14) | 11·81 (93) | 10·03 (139) | 1·17 (0·89 to 1·55) |
| Dose 2 | 10·83 (39) | 12·89 (74) | 0·90 (0·60 to 1·37) | 9·70 (82) | 10·03 (139) | 0·95 (0·71 to 1·26) |
| Dose 3 | 14·59 (32) | 14·41 (88) | 0·74 (0·48 to 1·15) | 15·24 (131) | 12·14 (231) | 0·85 (0·67 to 1·07) |
| **BBIBP-CorV** | |  |  |  |  |  |
| Dose 1 | 10·41 (17) | 16·05 (46) | 0·70 (0·39 to 1·27) | 13·83 (61) | 10·47 (93) | 1·40 (1·00 to 1·97) |
| Dose 2 | 8·76 (14) | 16·05 (46) | 0·61 (0·33 to 1·14) | 9·57 (51) | 10·47 (93) | 0·92 (0·65 to 1·31) |
| Dose 3 | 11·45 (13) | 17·26 (56) | 0·49 (0·25 to 0·94) | 14·80 (97) | 12·27 (168) | 0·90 (0·68 to 1·18) |
| **BNT162b2** |  |  |  |  |  |  |
| Dose 1 | 9·38 (21) | 9·74 (28) | 0·86 (0·44 to 1·69) | 9·24 (32) | 9·25 (46) | 0·71 (0·42 to 1·20) |
| Dose 2 | 12·48 (25) | 9·74 (28) | 1·26 (0·69 to 2·30) | 9·44 (31) | 9·25 (46) | 1·09 (0·73 to 1·69) |
| Dose 3 | 17·97 (19) | 11·19 (32) | 0·97 (0·51 to 1·82) | 16·63 (34) | 11·80 (63) | 0·65 (0·41 to 1·02) |
|  | **Age < 40 years** | | | **Age >= 40** | | |
| **Either vaccine** | |  |  |  |  |  |
| Dose 1 | 4·42 (39) | 5·26 (76) | 0·81 (0·54 to 1·22) | 31·34 (92) | 26·56 (137) | 1·19 (0·90 to 1·59) |
| Dose 2 | 5·09 (43) | 5·26 (76) | 0·84 (0·56 to 1·26) | 24·18 (79) | 26·56 (137) | 0·97 (0·73 to 1·30) |
| Dose 3 | 4·37 (27) | 4·56 (77) | 0·78 (0·48 to 1·27) | 29·44 (136) | 29·25 (242) | 0·84 (0·67 to 1·06) |
| **BBIBP-CorV** | |  |  |  |  |  |
| Dose 1 | 5·08 (23) | 5·46 (47) | 0·90 (0·53 to 1·51) | 36·12 (55) | 29·19 (92) | 1·27 (0·89 to 1·80) |
| Dose 2 | 3·93 (20) | 5·46 (47) | 0·70 (0·40 to 1·20) | 24·41 (45) | 29·19 (92) | 0·85 (0·59 to 1·23) |
| Dose 3 | 3·96 (18) | 4·53 (51) | 0·86 (0·46 to 1·58) | 29·22 (92) | 30·42 (173) | 0·82 (0·63 to 1·08) |
| **BNT162b2** |  |  |  |  |  |  |
| Dose 1 | 3·73 (16) | 4·96 (29) | 0·45 (0·21 to 0·95) | 26·19 (37) | 22·43 (45) | 0·93 (0·57 to 1·53) |
| Dose 2 | 5·28 (23) | 4·96 (29) | 1·05 (0·61 to 1·88) | 23·88 (34) | 22·43 (45) | 1·01 (0·62 to 1·66) |
| Dose 3 | 5·51 (9) | 4·63 (26) | 0·41 (0·17 to 0·98) | 29·89 (44) | 26·70 (69) | 0·83 (0·56 to 1·24) |

**Table S3. IRR (incidence rate ratio) and 95% CI by risk group (i.e., occurrence of health outcomes of interest in the preceding 12-month period before vaccination) for the primary outcome in the predefined risk interval of 1-21 days after exposure to vaccination. CI: confidence interval; pdays: person-days.**

|  | **With outcomes of interest in the preceding 1 year** | | |
| --- | --- | --- | --- |
|  | **Events in risk interval per 1M pdays (n)** | **Events in comparison interval per 1M pdays (n)** | **IRR (95% CI)** |
| **Either vaccine** |  |  |  |
| Dose 1 | 735·96 (45) | 531·37 (49) | 1·24 (0·80 to 1·93) |
| Dose 2 | 433·33 (26) | 531·37 (49) | 0·83 (0·50 to 1·37) |
| Dose 3 | 444·69 (35) | 423·18 (59) | 0·93 (0·60 to 1·44) |
| **BBIBP-CorV** |  |  |  |
| Dose 1 | 914·14 (27) | 540·72 (28) | 1·57 (0·88 to 2·80) |
| Dose 2 | 397·52 (13) | 540·72 (28) | 0·78 (0·39 to 1·55) |
| Dose 3 | 465·92 (23) | 436·68 (39) | 1·05 (0·61 to 1·82) |
| **BNT162b2** |  |  |  |
| Dose 1 | 569·46 (18) | 519·40 (21) | 1·08 (0·53 to 2·19) |
| Dose 2 | 476·23 (13) | 519·40 (21) | 0·77 (0·35 to 1·67) |
| Dose 3 | 408·97 (12) | 399·11 (20) | 0·83 (0·37 to 1·87) |
|  | **No Outcomes of Interest in the preceding 1 year** | | |
| **Either vaccine** |  |  |  |
| Dose 1 | 7·73 (78) | 8·77 (147) | 0·92 (0·68 to 1·24) |
| Dose 2 | 7·54 (82) | 8·77 (147) | 0·92 (0·69 to 1·22) |
| Dose 3 | 13·03 (126) | 10·91 (240) | 0·81 (0·64 to 1·03) |
| **BBIBP-CorV** |  |  |  |
| Dose 1 | 9·67 (44) | 10·52 (96) | 1·01 (0·69 to 1·47) |
| Dose 2 | 7·98 (43) | 10·52 (96) | 0·76 (0·52 to 1·10) |
| Dose 3 | 12·84 (85) | 11·94 (167) | 0·82 (0·61 to 1·09) |
| **BNT162b2** |  |  |  |
| Dose 1 | 6·14 (34) | 6·69 (51) | 0·59 (0·35 to 0·99) |
| Dose 2 | 7·11 (39) | 6·69 (51) | 1·06 (0·68 to 1·66) |
| Dose 3 | 13·43 (41) | 9·12 (73) | 0·68 (0·44 to 1·03) |


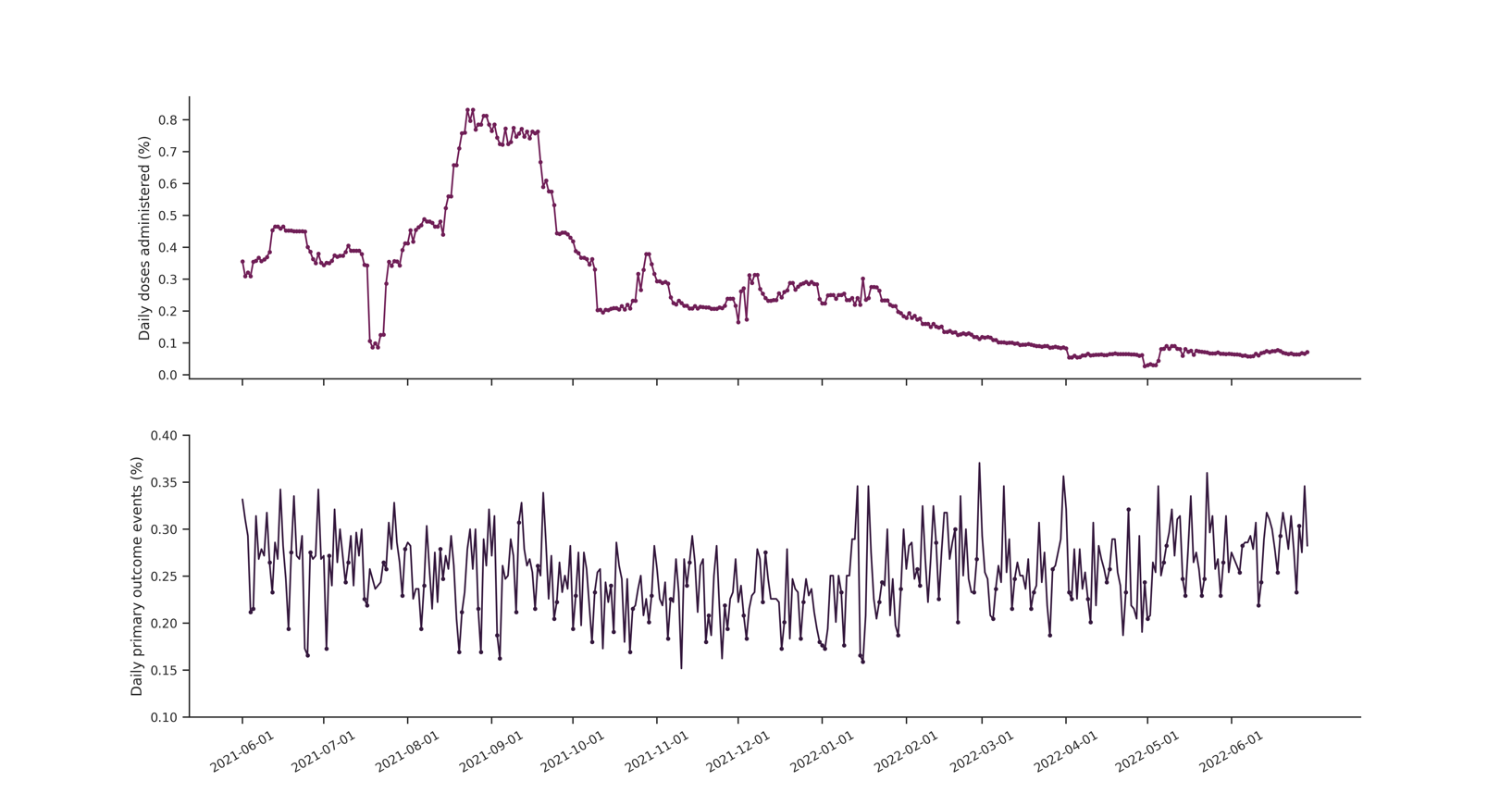


**Figure S1. Daily doses administered of both BBIBP-CorV and BNT16b2 vaccines during the study period (top); daily occurrence of primary (composite) outcome events over the study period (bottom). Markers (dots) in the bottom plot show days that correspond to weekend days.**
