# Supplementary material for: COVID-19 vaccination and major cardiovascular and haematological adverse events in Abu Dhabi: retrospective cohort study
Source: Nat Commun. 2024 Jun 28;15:5490. doi: 10.1038/s41467-024-49744-6 (PMC11214614; doi:10.1038/s41467-024-49744-6)
Supplement: Supplementary file 3 — Reporting Summary [file 41467_2024_49744_MOESM3_ESM.pdf]

nature portfolio

Corresponding author(s): Maaz Shaikh

Last updated by author(s): May 4, 2024

Reporting Summary

Nature Portfolio wishes to improve the reproducibility of the work that we publish. This form provides structure for consistency and transparency in reporting. For further information on Nature Portfolio policies, see our Editorial Policies and the Editorial Policy Checklist.

Statistics

For all statistical analyses, confirm that the following items are present in the figure legend, table legend, main text, or Methods section.

n/a	Confirmed	
☐	☒	The exact sample size (*n*) for each experimental group/condition, given as a discrete number and unit of measurement
☐	☒	A statement on whether measurements were taken from distinct samples or whether the same sample was measured repeatedly
☐	☒	The statistical test(s) used AND whether they are one- or two-sided<br>*Only common tests should be described solely by name; describe more complex techniques in the Methods section.*
☐	☒	A description of all covariates tested
☐	☒	A description of any assumptions or corrections, such as tests of normality and adjustment for multiple comparisons
☐	☒	A full description of the statistical parameters including central tendency (e.g. means) or other basic estimates (e.g. regression coefficient) AND variation (e.g. standard deviation) or associated estimates of uncertainty (e.g. confidence intervals)
☐	☒	For null hypothesis testing, the test statistic (e.g. *F*, *t*, *r*) with confidence intervals, effect sizes, degrees of freedom and *P* value noted<br>*Give P values as exact values whenever suitable.*
☒	☐	For Bayesian analysis, information on the choice of priors and Markov chain Monte Carlo settings
☒	☐	For hierarchical and complex designs, identification of the appropriate level for tests and full reporting of outcomes
☒	☐	Estimates of effect sizes (e.g. Cohen's *d*, Pearson's *r*), indicating how they were calculated

Our web collection on statistics for biologists contains articles on many of the points above.

Software and code

Policy information about availability of computer code

Data collection	No software was used as not additional data was collected specifically for this study. We used de-identified data from the Abu Dhabi Health Information Exchange (HIE), known as “Malaffi”, to identify COVID-19 vaccine exposure. The Malaffi HIE receives data from all licensed facilities within the Emirate of Abu Dhabi, and to date, it contains historical records from over 8 million individuals. This includes demographic information (including nationality), diagnosis codes, medications, immunization, laboratory results, in-hospital mortality information, and other procedures associated with patients’ visits to the care facilities. Most notably, these data are linked to vaccination data (including vaccine type, date, dose number, and site of administration), and the severe acute respiratory syndrome coronavirus 2 (SARS-CoV-2) infection data for all people in Abu Dhabi.
Data analysis	Custom Python and R scripts have been used to conduct the data processing and statistical analyses.

For manuscripts utilizing custom algorithms or software that are central to the research but not yet described in published literature, software must be made available to editors and reviewers. We strongly encourage code deposition in a community repository (e.g. GitHub). See the Nature Portfolio guidelines for submitting code & software for further information.

Data

Policy information about availability of data

All manuscripts must include a data availability statement. This statement should provide the following information, where applicable:

- Accession codes, unique identifiers, or web links for publicly available datasets
- A description of any restrictions on data availability
- For clinical datasets or third party data, please ensure that the statement adheres to our policy

According to Abu Dhabi Department of Health regulations, individual-level data cannot be shared openly. Specific requests for remote access to de-identified data should be referred directly to DOH, Research committee.

Research involving human participants, their data, or biological material

Policy information about studies with human participants or human data. See also policy information about sex, gender (identity/presentation), and sexual orientation and race, ethnicity and racism.

Reporting on sex and gender	In the study design phase, gender was a primary consideration, leveraging routinely collected data from electronic health records across multiple care facilities. The investigation aimed to discern potential gender-dependent associations between the exposure and outcomes of interest. The dataset for our analysis encompasses 1,312,505 individuals, with a breakdown of 966,359 males, 345,722 females, and 424 individuals with unknown (or not reported) gender.<br>Our approach to gender classification relied on the information routinely recorded as part of clinical practice. Ethical approval was secured to conduct and report the results of this study. We affirm that individual-level data, including disaggregated sex and gender information, has been appropriately handled, with due consideration given to privacy and consent. In instances where such data was not available or consent for sharing was not obtained, this is explicitly stated.
Reporting on race, ethnicity, or other socially relevant groupings	Originally considering race as a potential confounding factor based on insights from existing literature, we encountered limitations in the reliable recording of race within electronic healthcare records. Consequently, we opted to use ancestry or region of origin as a more feasible and robust classification variable in our investigation. This variable was chosen to reflect the genetic heritage of individuals and was categorized according to the regions reported by the World Health Organization, based on their country of origin.<br>Acknowledging the importance of addressing potential confounding variables, our analytical approach involved conducting analyses within strata defined by region of origin and other pertinent variables.
Population characteristics	Our study reports the results of the analyses performed on health data records from 1,312,505 individuals aged 12 years or older with at least one exposure of interest during our study period. Median (interquartile range) is reported at 35 (27-42), with less than one third of the individuals being 40 years or older. Less than 1% of individuals had a reported event of interest within one year preceding the exposure.
Recruitment	No recruitment has been conducted.
Ethics oversight	Abu Dhabi Health Research and Technology Ethics Committee of the Abu Dhabi Department of Health.

Note that full information on the approval of the study protocol must also be provided in the manuscript.

Field-specific reporting

Please select the one below that is the best fit for your research. If you are not sure, read the appropriate sections before making your selection.

☒ Life sciences ☐ Behavioural & social sciences ☐ Ecological, evolutionary & environmental sciences

For a reference copy of the document with all sections, see nature.com/documents/nr-reporting-summary-flat.pdf

Life sciences study design

All studies must disclose on these points even when the disclosure is negative.

Sample size	No sample-size calculation was performed at the start of the study. Sample sizes were determined according to the number of exposures occurred during the study period considered. The study period coincided with the the COVID-19 vaccination rollout program, which maximizes the number of individuals included in this investigation. Sample size calculation were conducted retrospectively as part of the peer review process. Based on our analysis, taking into consideration the incidence rate observed in terms of number of participants (i.e., n = 1,312,505), we determined with 80% power at a significance level of 0.05, sample sizes of (approximately) 1.3 million and 0.5 million participants would be required to detect a 30% and 50% increase in risk, respectively. Hence, with our sample size, the study can detect a risk increase of around 30%.
Data exclusions	Attending to the study design, we excluded records from individuals with no exposures recorded during the study period. We also excluded individuals with no recorded year of birth, as we were not able to determine the age of the individual at the time of the exposure.
Replication	No experiments were conducted in this study. This was a retrospective study using previously captured data. Analysis was verified for

Replication	accuracy.
Randomization	Randomization is not relevant to our study, as it relies on observational and a quasi-experimental design rather than randomization of participants into distinct groups. We adopted a vaccine surveillance methodology, known as rapid cycle analysis, which was developed originally to monitor vaccine safety, i.e., it has been used in vaccine safety studies to quickly analyze and assess real-time data and the rapid detection of any unexpected patterns or trends on adverse events following immunization. In these studies, the rate of adverse events in individuals before vaccination (baseline) to the rate after vaccination is compared. Any significant increase in adverse events post-vaccination may suggest a potential association. The analyses are well-adjusted for calendar date, which avoids biases that arise from day-to-day variation in health services. In addition, they are not confounded by time-stable characteristics during the study period, such as gender, ethnicity, and chronic conditions, which can be controlled for.
Blinding	Blinding is not directly applicable as in traditional clinical trials. Our study involves the continuous monitoring and analysis of real-world data, i.e., electronic health records.

Reporting for specific materials, systems and methods

We require information from authors about some types of materials, experimental systems and methods used in many studies. Here, indicate whether each material, system or method listed is relevant to your study. If you are not sure if a list item applies to your research, read the appropriate section before selecting a response.

Materials & experimental systems

n/a	Involved in the study
☒	☐ Antibodies
☒	☐ Eukaryotic cell lines
☒	☐ Palaeontology and archaeology
☒	☐ Animals and other organisms
☐	☒ Clinical data
☒	☐ Dual use research of concern
☒	☐ Plants

Methods

n/a	Involved in the study
☒	☐ ChIP-seq
☒	☐ Flow cytometry
☒	☐ MRI-based neuroimaging

Clinical data

Policy information about clinical studies

All manuscripts should comply with the ICMJE guidelines for publication of clinical research and a completed CONSORT checklist must be included with all submissions.

Clinical trial registration	This study was not registered with clinicaltrials.gov
Study protocol	This study was approved by the Abu Dhabi Health Research and Technology Ethics Committee (approval number DOH/CVDC/2022/1466) of the Abu Dhabi Department of Health(s).
Data collection	This study was conducted in Abu Dhabi. People were considered eligible for inclusion in this study if they had received at least one vaccine dose, were at least 12 years old (at the time of vaccination), and were admitted to hospital or had an ER visit with the outcome of interest (i.e., follow-up study period) between 1 June 2021 and 30 June 2022.
Outcomes	The outcomes in this study are selected cardiovascular and haematological conditions occurring post administration of COVID-19 vaccine. These included acute myocardial infarction (AMI), non-haemorrhagic stroke (NHS), haemorrhagic stroke (HS), myocarditis or pericarditis (M/P), disseminated intravascular coagulation (DIC), pulmonary embolism (PE), and venous thromboembolism (VTE). The outcomes were identified as hospital admissions (inpatients) or ER visits due to an event of interest recorded within the study period. We defined the primary outcome of this study as a composite event represented by the first hospitalization or ER visit in each day for any of the selected adverse events during the study period. Secondary outcomes of the study were outcome-specific for each one of the seven CVDH adverse events mentioned above.

Plants

Seed stocks	Not applicable.
Novel plant genotypes	Not applicable.
Authentication	Not applicable.
